# Detecting Identity by Descent and Homozygosity Mapping in Whole-Exome Sequencing Data

**DOI:** 10.1371/journal.pone.0047618

**Published:** 2012-10-11

**Authors:** Zhong Zhuang, Alexander Gusev, Judy Cho, Itsik Pe'er

**Affiliations:** 1 Department of Computer Science, Columbia University, New York, New York, United States of America; 2 Center for Computational Biology and Bioinformatics, Columbia University, New York, New York, United States of America; 3 Section of Digestive Diseases, Department of Internal Medicine, Yale University School of Medicine, New Haven, Connecticut, United States of America; Max Planck Institute for Evolutionary Anthropology, Germany

## Abstract

The detection of genetic segments of Identical by Descent (IBD) in Genome-Wide Association Studies has proven successful in pinpointing genetic relatedness between reportedly unrelated individuals and leveraging such regions to shortlist candidate genes. These techniques depend on high-density genotyping arrays and their effectiveness in diverse sequence data is largely unknown. Due to decreasing costs and increasing effectiveness of high throughput techniques for whole-exome sequencing, an influx of exome sequencing data has become available. Studies using exomes and IBD-detection methods within known pedigrees have shown that IBD can be useful in finding hidden genetic candidates where known relatives are available. We set out to examine the viability of using IBD-detection in whole exome sequencing data in population-wide studies. In doing so, we extend GERMLINE, a method to detect IBD from exome sequencing data by finding small slices of matching alleles between pairs of individuals and extending them into full IBD segments. This algorithm allows for efficient population-wide detection in dense data. We apply this algorithm to a cohort of Crohn's Disease cases where whole-exome and GWAS array data is available. We confirm that GWAS-based detected segments are highly accurate and predictive of underlying shared variation. Where segments inferred from GWAS are expected to be of high accuracy, we compare exome-based detection accuracy of multiple detection strategies. We find detection accuracy to be prohibitively low in all assessments, both in terms of segment sensitivity and specificity. Even after isolating relatively long segments beyond 10cM, exome-based detection continued to offer poor specificity/sensitivity tradeoffs. We hypothesize that the variable coverage and platform biases of exome capture account for this decreased accuracy and look toward whole genome sequencing data as a higher quality source for detecting population-wide IBD.

## Introduction

The identification of co-inherited genomic regions, referred to as being Identical by Descent (IBD) has been used to find hidden relatedness [1], make inferences regarding population genetics [2], detect association [3], and correct errors in genotyping and phasing [4]. IBD falls into three categories: IBD = 0, IBD = 1, and IBD = 2. Siblings that inherit both of the same chromosomes from both parents are IBD = 2. Siblings that inherit one chromosome are IBD = 1. Siblings that do not inherit the same chromosome are IBD = 0. The IBD being used later within this work looks at sites that are homozygous and identical between both individuals on both chromosomes, thus a subset of IBD = 2 individuals. The frequency of IBD segments depends on the population, and scales quadratically with the cohort size [5]. Discovering such segments can be done efficiently by looking for short, exact matches between individuals and extending them to identify long, nearly identical segmental sharing that is indicative of such relatedness. We previously presented GERMLINE as a robust algorithm to analyze genotype array data and identify segmental sharing indicative of recent common ancestry between pairs of individuals [5]. The paradigm of exact-segment extension has also been employed by other methods. Some array-based IBD discovery methods use haplotype sampling and to detect large segments of identity by state [4], [5], [6]. Others employ the Hidden Markov Models (HMM) to find nonrandom increases of alleles indicating probability of IBD [2], [7], [8]. Given the numerous developments in array-based IBD detection, there is hope that advances can be made in detecting IBD directly from next-generation sequence data.

Exome sequencing by hybrid capture is the current assay of choice to efficiently sequence the coding regions of the genome. These developments have enabled efficient discovery of highly penetrant variants that are too rare to be detected by SNP-array studies [9]. Decreasing costs and increasing effectiveness of high throughput techniques for whole-exome sequencing has ushered an influx in whole-exome sequencing data. Large numbers of exomes are being made available by studies such as the Thousand Genomes Project [10], with many IBD segments expected between the millions of pairs of samples from such studies. The ability to detect IBD in these datasets and supplement IBD analysis of SNP arrays will become critical as more data is obtained.

**Figure 1:**
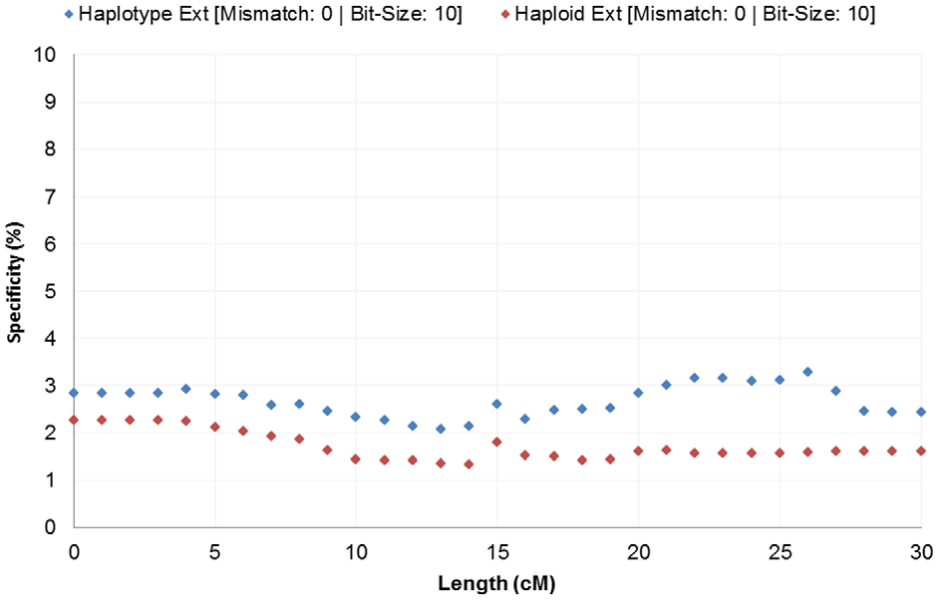
Exome specificity with varying segment length. Exome sequencing IBD data is shown to have low specificity with GWAS IBD data. Even at lengths of 30 cM and with stringent IBD segment generation parameters, the specificity remains low at under 3%.

**Figure 2:**
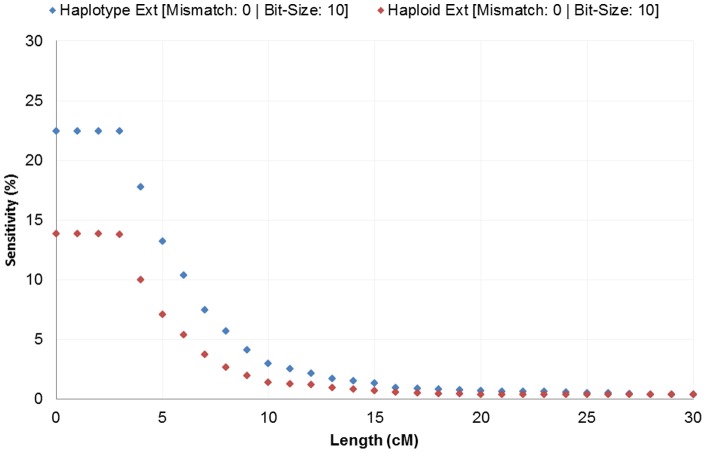
Exome sensitivity with varying segment length. Exome sequencing IBD data is shown to have low sensitivity with GWAS IBD data. The sensitivity decreases as the threshold increases for allowed segment length.

**Figure 3:**
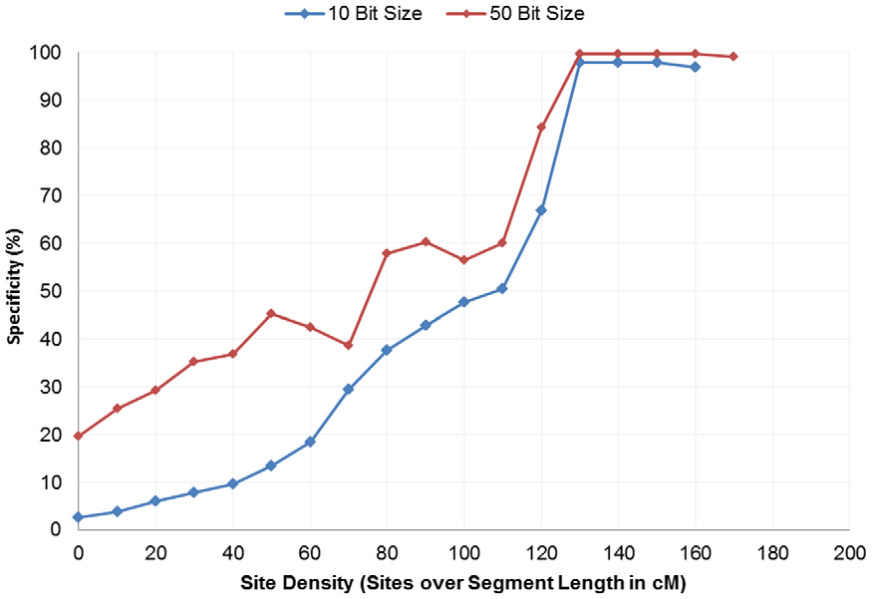
Exome specificity with varying exome site density. We see an increase in specificity of exome generated IBD segments in detecting data verified by GWAS IBD as we increase the threshold of site density.

**Figure 4:**
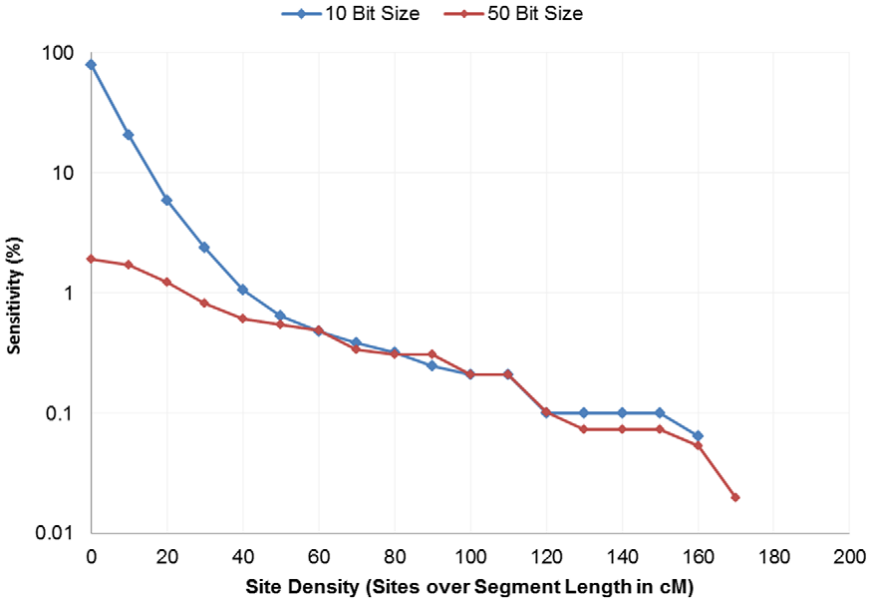
Exome sensitivity with varying exome site density. We see a decrease in sensitivity of exome generated IBD segments in their ability to cover the segments discovered from GWAS IBD as we increase the threshold of site density.

**Figure 5:**
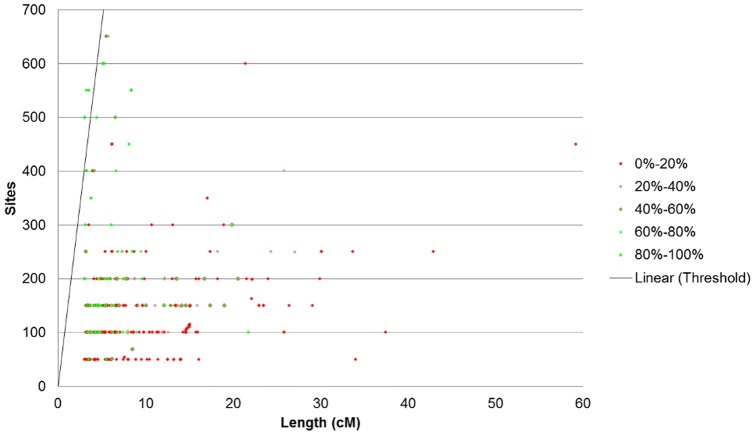
Exome specificity with varying sites and segment length. Exome specificity as a function of both with varying site frequency and segment length. Using specificity data generated for the 50 bit size data seen, we examine here the relationship between the specificity and the site density. Areas of high specificity (color coded green) are concentrated in the portion of the graph where segments demonstrate site density, with high site frequency and low length. Based on the figure, a qualitative observation can be made about how increased site density correlates with increased specificity.

IBD segments have been used effectively in conjunction with known pedigree information for related individuals who share a specific highly-penetrant disease. Intersecting the search for novel variation, coding changes and IBD segments across multiple affected family members can successfully narrow down the putative causal mutation to a single variant. In particular, this technique was proven useful in identifying gene candidates for Miller syndrome by seeking genes with IBD patterns indicating recessive inheritance [11]. However, the methods used to detect IBD in pedigrees with whole-exome data are currently based on an exhaustive pairwise Hidden Markov Model and are not efficient enough to handle population-wide cohorts where the relatives are unknown and the pedigree unavailable. Our work with GERMLINE aims to use such data to efficiently infer IBD and make population-wide analysis possible.

**Table 1:** Concordance between GWAS IBD and whole-exome sequencing data.

Call Quality^ae^		Random^d^	Genotype extension	Haplotype extension	Haploid extension
**Novel Sites** b	**0**	*15.65%*	*26.45%*	*33.16%*	*63.95%*
	**10**	*15.65%*	*44.91%*	*54.87%*	*82.32%*
	**20**	*15.98%*	*66.66%*	*75.51%*	*93.07%*
	**30**	*16.43%*	*78.17%*	*81.58%*	*95.08%*
	**40**	*17.10%*	*82.01%*	*87.10%*	*99.07%*
**Annotated Sites** c	**0**	*71.46%*	*94.46%*	*95.02%*	*96.97%*
	**10**	*71.46%*	*97.78%*	*97.89%*	*98.89%*
	**20**	*71.47%*	*98.62%*	*98.67%*	*99.16%*
	**30**	*71.48%*	*99.05%*	*99.08%*	*99.41%*
	**40**	*71.50%*	*99.22%*	*99.20%*	*99.40%*

a Minimum genotype quality threshold for variant call.

b Sites not present in dbSNP 130.

c Sites present in dbSNP 130.

d Expected concordance measured across all loci.

e 800000 genotype sites, 155327 exome sites, and 28145 filtered exome sites are compared.

Here, we develop a method and tool for the discovery of IBD segments in whole-exome sequencing data from large cohorts of reportedly unrelated individuals. We do so by improving on the current GERMLINE software through extension methods and use it to process whole-exome sequencing data. We consider samples from an Ashkanazi Jewish (AJ) cohort, where IBD is prevalent [12] facilitating a large number segments to be analyzed even amid constraints on sample size. These individuals are sampled from a larger cohort of Crohn's Disease (CD) cases, for which both whole-exome sequencing data and GWAS data has been obtained, facilitating cross-comparisons of IBD segments inferred from either data. We seek to analyze two challenges: (1) The ability to consistently detect the same IBD segments in exome and SNP-array datasets of the same samples and (2) the extent of each such dataset to contain information necessary to reject IBD segments detected in its counterpart or concord with them.

## Materials and Methods

### Crohn's Disease dataset

Exome sequencing was performed using the Illumina GA2 platform and Agilent SureSelect hybrid capture. SNP array data was obtained using the Illumina 1 M platform. Samples are broadly consented 49 Crohn's Disease cases of Ashkenazi Jewish ancestry from New York and Connecticut.

### Evaluating segment overlap

We focused on measuring overlap in IBD segments from array data and whole-exome sequencing data using GERMLINE. Briefly, GERMLINE operates by breaking up genetic data into slices of equal size or windows, and then uses a dictionary-based method to identify exact matches between individuals. It extends these initial matching slices (seeds) into long and nearly-exact matches. While GERMLINE had been designed for SNP-array data, we have modified it to process whole-exome sequencing data.

We have observed previously that long IBD segments detected from SNP-arrays have close to 100% specificity. Consistent with this assumption, we categorize the array-based segments as ground truth and measure relative specificity and sensitivity for segments detected in the exome data. For any pair of samples *i, j* we identify IBD segments in the array data where each segment is an interval of physical start and end (*a, b*), and all intervals comprise a set **IBD**_array_(i,j)_._ We construct a similar set from the exome data, **IBD**_exome_(i,j). Given an interval π from **IBD**, we can calculate the length of overlap between that interval and a list of other intervals as α(*π*, **IBD**'). We can also calculate the length of the interval π as *length*(π). Specificity, as measured across all pairs (*i, j*), is then calculated as:
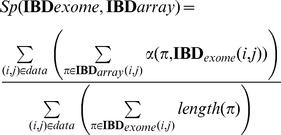



This measures the proportion of whole-exome sequencing data produced that is consistent with array data. Similarly, we calculate sensitivity across all pairs (*i, j*) as
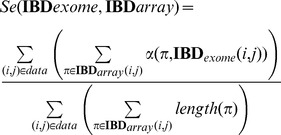



This measures the proportion of array-based segments detected by the exome data. Both measures of overlap were parameterized with respect to the allowed mismatch error and bit size: the size of each slice used for the exact matching seed. The parameters were optimized using grid-search.

### Evaluating concordance of IBD across array and whole-exome data

To estimate the absolute quality of array-based IBD segments, we also measured the concordance of exome-only sites that were not used in segment detection. Concordance is defined above where at a site, two individuals are both homozygous and identical. If there is a true IBD segment two between individuals, any homozygous sites should be concordant between the two (with some allowed calling error), whereas two individuals with IBD  = 0 would exhibit concordance based on the site allele frequency. Therefore, we use concordance as a measure of likelihood that the detected segment is truly IBD. To avoid counting the majority of the genome which is homozygous for the reference allele, we calculate concordance only over sites that are variant in at least one sample. We also examine individual sites of concordance to identify IBD rather than looking for regions that are homozygous. Alternatively, one can think of concordance as the accuracy of predicting an unseen homozygous variant in one individual conditional on the presence of an IBD segment and an observed homozygous variant in the other individual.

Formally, for an IBD segment between samples (*i,j*) along the interval (*a,b*) we calculate whether locus *n* between *a* and *b* is homozygous in either *i* and *j* and carries the variant allele in either with indicator function 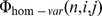. Similarly, we calculate whether the location has identical alleles at such sites with indicator 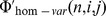. For every interval (*a,b*) in **IBD_array_**_ (***i, j***),_ we then compute concordance as:
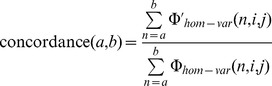
and average this measure across all intervals. As we expect no discordant alleles within true IBD segments, concordance of 100% signifies that all the exome sites in the raw data verified the matches detected through array-based IBD, while low concordance indicates low quality of IBD or low-quality in calling the variants (causing true heterozygous sites to be miscalled as homozygous). For comparison, we can estimate expected baseline average concordance given the carrier counts for site *n*: 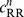, the number of homozygous reference carriers; and 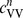, the number of homozygous variant carriers. The probability of observing a site where both samples are homozygous and at least one is a homozygous variant given sample size *s* is:



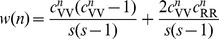
then the genome-wide average probability of observing the second homozygous variant carrier given that one has been observed, weighted by *w(n)* is:



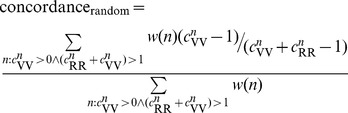



We use the homozygote frequencies directly to avoid biases from sites not in Hardy-Weinberg Equilibrium. So as to account for difference in call quality, we measured both types of concordance over a range of genotype quality thresholds (GQ).

The GERMLINE algorithm is particularly susceptible to different algorithms for extending from the exact initial seeds, which leads us to measure concordance under three separate metrics. All metrics are dependent for a mismatch parameter *m* which simply measures the number of sites that have no shared alleles. *Genotype extension* allows extension through a slice if only the sites that are mutually homozygous between the pair of samples have less than *m* mismatches. Ignoring any heterozygotes, this approach is entirely agnostic of haplotype phase but may ignore useful haplotype information. *Haplotype extension* allows extension through a slice if any of the four haplotype pair combinations have less than *m* mismatches. This metric is aware of haplotypes but allows for phase switching across slices. *Haploid extension* is identical to haplotype extension but does not allow for switching across slices, requiring a single phased haploid chromosome to have fewer than *m* mismatches per slice through the entirety of the segment. In practice, we have found this order of extension criteria to be increasingly restrictive.

## Results

Overall, we measured low signal within IBD generated from whole exome IBD detection. Detection had limited specificity at 2.4% and low sensitivity at less than 0.3% (Figure 1 and Figure 2). Although detection in whole-genome data increases in accuracy with length, we see no such trend in the exome-based detection. Even long segments of 30 cM detected in exomes had relatively small overlap (2.43% specificity and 0.36% sensitivity) with array data. Low accuracy was observed with no mismatches allowed and consistent across all extension metrics. Limiting exome-detected segments to those with higher site density (measured as SNPs/cM), the specificity increased significantly, with a corresponding decreasing sensitivity. At a threshold of at least 130 sites per cM the overlap specificity had increased to 97.82% and 99.77% for the 10 bit and 50bit seeds respectively (Figure 3). The sensitivity had decreased to 0.10% and 0.07% respectively (Figure 4). To further understand the nature of site density and its effect on IBD detection, we plot exome specificity as a function of both varying site frequency and segment length. We see that areas of high specificity (color coded green) are concentrated in the portion of the graph where segments demonstrate site density (Figure 5).

We estimated the accuracy of IBD detected from the array by measuring homozygous variant concordance (Methods) over exome-only sites (Table 1) that have been confirmed in dbSNP (v130) or are novel. With default GERMLINE parameters (genotype extension), we measured a concordance of 94.46% over dbSNP sites and 26.46% in novel sites (compared to random concordance of 71.46% and 15.65% respectively). After filtering for high-quality calls (GQ>40), a concordance of 99.22% was achieved on the dbSNP sites and 82.00% on novel sites. By using haploid extension we were able to increase annotated concordance to 96.97% on dbSNP sites and to 63.95% for the novel sites without filtering for genotype quality; and to 99.40% and 99.07%. High levels of concordance between GWAS IBD and exome sequencing data reflect the high quality of IBD detection.

## Discussion

With steadily decreasing costs, collecting whole-exome data from thousands of individuals has become a tractable task and we can expect GWAS-sized exome studies to soon become ubiquitous. Early findings from family-based exome studies have demonstrated the power of using IBD segments to localize potential causal coding variants. We have detailed a method for efficiently detecting such segments in large-scale exome data and investigated the feasibility of our approach in characterizing such segments accurately and completely. The method was tested in a unique data-set of Ashkenazi Jewish samples, where we expect a relatively high number of IBD sharing. In particular, data collected from individuals with whole-exome sequencing and whole-genome array genotyping platforms allowed us to empirically measure IBD detection accuracy. We've found that the use of this method on whole exome-sequencing data shows little overlap with IBD segments detected from GWAS data. Based on these measurements, we conclude that the amount of high quality IBD data that can be generated purely from whole-exomes is inadequate. While filtering based on marker density and seeking out very long segments offers marginal improvements to accuracy, the significant proportion of entirely false-positive segments remains prohibitive.

Based on these findings, we hypothesize that efficient allele-based IBD detection has limited utility in whole-exomes due to the nature of such data. In whole-genome IBD detection, uniform coverage of SNPs scattered evenly throughout the genome allows for accurate detection of IBD segments. There, genetic distance of an inferred segment has been shown to strongly correlate with detection accuracy, where accuracy increases with underlying segment length. Exome sequencing, however, contains highly irregular density of read targets and therefore coding variants. Within exonic regions, we have deeply ascertained rare and common variants that may aide in segment detection. However, because rare variants are likely to be heterozygous and difficult to phase and IBD detection relies either on homozygous sites or well-phased haplotypes, their contribution will be limited. Outside of exonic regions (and where off-target reads are not considered) variant calling is largely non-existent, making IBD detection more difficult. In addition, the exome platform may contain significant reference-allele bias due to exome capture and alignment [13], which further reduces the information available for accurate detection. Recently, studies have applied these same IBD detection algorithms to whole-genome sequence and yielded very high detection accuracy [14]. Thus, we suspect that it is this difference in targets between the platforms that causes the detection in whole-exome sequence to be weak. Thus, the high variance in marker density across exome data and the decreased allelic diversity within exons no longer allows for such an assumption. In particular, we have found a significant number of long exonic regions of complete identity between pairs of individuals that are revealed to be merely identical by state from whole-genome data. Such segments would be impossible to distinguish from true segments without complex population-based priors on haplotype frequency from separate a control group. We consider the possibility of GERMLINE's false positive rates affecting our result; However, false positive rates, liberally estimated to be 7.4×10^−6^, would not account for the lack in specificity seen within the data [7]. What would improve detection would be to have more uniform variants. Potentially, we could have more even coverage by using off target reeds.

While the segments detected from exomes pose problems of accuracy, we do find that segments identified in the whole-genome data are useful as predictors of allelic similarity in unobserved variants. Specifically, we find that novel, high-quality variants identified in one individual of an array-based IBD segment pair is nearly guaranteed to be seen in the other. As whole exome data becomes more prevalent, this high rate of concordance suggests the possibility of accurately inferring whole exome data from presently available GWAS IBD data.

Further studies should account for the potential challenges of exome data by modifying IBD detection algorithms to better adapt to the density variations and decreased allelic diversity within exons. Such modification may go beyond simply filtering based on SNP density to better detect IBD. Furthermore, we look toward whole genome sequencing as a means of providing a higher quality data with less variation in site density. Given data with a clear ground truth, like high quality whole genome sequencing data, IBD performed on merged exome sequencing and genotype data could definitely help determine utility of running IBD on the two datasets in conjunction.
